# The Association between the Availability of Sugar-Sweetened Beverage in School Vending Machines and Its Consumption among Adolescents in California: A Propensity Score Matching Approach

**DOI:** 10.1155/2010/735613

**Published:** 2010-10-03

**Authors:** Lu Shi

**Affiliations:** Department of Health Services, UCLA School of Public Health, 650 Charles E. Young Drive S., 61-253 CHS, Los Angeles, CA 90095, USA

## Abstract

There is controversy over to what degree banning sugar-sweetened beverage (SSB) sales at schools could decrease the SSB intake. This paper uses the adolescent sample of 2005 California Health Interview Survey to estimate the association between the availability of SSB from school vending machines and the amount of SSB consumption. Propensity score stratification and kernel-based propensity score matching are used to address the selection bias issue in cross-sectional data. Propensity score stratification shows that adolescents who had access to SSB through their school vending machines consumed 0.170 more drinks of SSB than those who did not (*P* < .05). Kernel-based propensity score matching shows the SSB consumption difference to be 0.158 on the prior day (*P* < .05). This paper strengthens the evidence for the association between SSB availability via school vending machines and the actual SSB consumption, while future studies are needed to explore changes in other beverages after SSB becomes less available.

## 1. Introduction

Health researchers and public health activists found school the environment to be an important determinant of eating and drinking behaviors among children [1, 2]. Competitive foods, that is, foods and beverages sold from vending machines, school stores, and so forth, remain a prevalent health risk for school-age children [3–7]. In recent years, efforts have been focused on taxing sugar elements in the sugar-sweetened beverage (SSB) or sugar itself. For example, California bill SB677, a law passed in 2003, bans soda from elementary and middle schools and limits soda sales in high schools during school hours. However, there remains considerable controversy over how much the increase in the consumption of SSB has contributed to the increase in childhood obesity and how much limiting the SSB sales at schools could decrease the soda intake and the body weight [8, 9]. 

This paper uses population survey data to examine the magnitude of the association between the availability of sugar-sweetened beverage from schools' vending machines and the amount of SSB consumption among California's adolescents, while controlling for sociodemographic and behavioral confounders [10]. Specifically, by estimating how much the availability of SSB through school vending machines independently predicts the SSB consumption, this study provides a benchmark for future evaluation of SSB interventions among younger populations.

## 2. Method

The dataset used in this study is the adolescent sample of 2005 California Health Interview Survey (CHIS). CHIS is a biennial population health survey based on telephone interviews, and its adolescent sample is conducted with adolescents living in sampled households [11]. The 2005 CHIS adolescent sample asked the respondent whether his or her school has SSB available at vending machines, which is the key independent variable of this study. The survey also asked the respondent how many servings of SSB he or she had on the prior day, which is used in this study as the outcome variable.

To address the selection bias that might occur with cross-sectional survey data, this study uses propensity score matching [12] to create a control group (adolescents whose school did not have SSB available through vending machines) that is similar to the exposure group (adolescents whose school did have SSB available through vending machines) in all observed confounding predictors of SSB intake. In this context, the predicted probability of attending a school that has SSB through vending machines (the propensity score) is estimated through a logistic regression. Each individual in the exposure group is then compared with control group members that have a close propensity score, and their differences in the outcome variable (in our case, the number of SSB the adolescent had on the prior day) are summed to give an overall difference, which indicates whether the exposure variable (SSB availability through schools' vending machines) is significantly associated with the outcome variable. 

In this analysis, only 42.6% of the sample are control cases (i.e., the adolescent's school does not have soda in its vending machines), which means that propensity score matching methods like nearest neighbor and radius matching could mean throwing away a lot of observations and increasing the variance of the estimator [13]. Thus, we use matching methods that make use of all observations in implementing the propensity score matching: propensity score stratification and kernel-based matching [14]. Stratification matching, as implemented in this study, stratifies the sample into five strata such that within each stratum, treated and control units have the same average propensity score. The average treatment effect is calculated by averaging the between-group outcome differences over the five strata. Kernel-based matching, on the other hand, compares each exposure case with a weighted sum of all control cases, with the weights inversely related to the propensity score difference between the exposure case and the control case. These two matching methods were implemented by the user-written commands of atts and attk in STATA 10, while the propensity score was computed by user-written STATA program of pscore.ado. The predictors we use in the probit regression include the adolescent's gender, age (and a quadratic term of age squared), race/ethnicity, parental education, household income level (at or above the federal poverty level), and whether the adolescent attended a public school.

## 3. Results

Of all 4029 adolescents who responded to the 2005 CHIS survey, 46 gave no answer or said “don't know,” 2285 reported that their schools had SSB available through the vending machines, and 1698 said that their schools did not have SSB through the vending machines. Table 1 lists the descriptive characteristics of the 3983 adolescents who gave a yes or no answer to the SSB availability question. The predictors were then used in a probit regression (Table 2) to produce a propensity score that represents the predicted probability of being exposed to an SSB-selling vending machine at school. The entire sample was then divided into nine blocks according to different propensity score values, and *t*-tests were run within each block to check if exposure and control cases were similar to each other in all confounding variables. These *t*-tests show that the differences in predictors between the two groups were not significant, which means that the propensity score used here successfully created a control group comparable to the exposure group. Table 3 shows the means of propensity scores in the exposure group and the control group for each block, while Table 4 shows the means of SSB consumption in the exposure group and the control group for each block.

Table 5 shows the results of the two propensity score-matched comparisons. Propensity score stratification shows that adolescents who had access to SSB through their school vending machines consumed 0.181 more drinks of SSB than those who did not (*P* < .05). Kernel-based propensity score matching shows the SSB consumption difference to be 0.159 on the prior day (*P* < .05).

## 4. Conclusion

With a population-representative large sample, this study strengthened the evidence for the association between SSB availability via school vending machines and the actual SSB consumption. The use of propensity score matching, a method designed to address the selection bias, further showed that the SSB availability at school vending machines and the SSB consumption have an independent and unambiguous association. Recent evidence shows that both the SSB consumption and the childhood obesity declined after California's ban on soda sales at schools in 2003 [15], and this study helps us understand a possible mechanism behind these phenomena.

Ludwig et al. [16] estimated from a longitudinal sample of younger adolescents that for each additional serving of consumed SSB, both body mass index (BMI) (mean 0.24 kg/m^2^; *P* = .03) and frequency of obesity (odds ratio 1.60; *P* = .02) increased, after being adjusted for anthropometric, demographic, dietary, and lifestyle variables. If one additional serving of SSB per day increases the odds of being obese by 60%, then our estimated effect of SSB availability through school vending machines on daily SSB consumption, 0.181 or 0.159 serving, is not an ignorable factor in childhood obesity prevention. Our descriptive analysis showed that the average consumption of SSB on the prior day was 1.09 serving, which means that on average the exposure to SSB from school vending machines could account for around one sixth of the daily SSB consumption among adolescents aged 12–17. If this might seem like a larger effect than what was shown by earlier studies of elementary school students (e.g., Fernandes [6]), this might be due to the fact that adolescents are more likely to buy beverage from school vending machines than children under 12. Thus, banning SSB sales at schools has a larger effect among adolescents than among younger children. 

The broader significance of reducing children's exposure to SSB lies beyond childhood obesity prevention. Some of the SSBs could cause mental disorders among children and adolescents via their caffeine component [17],and SSB consumption is also associated with dental caries among children [18]. Moreover, as adolescence is a time when taste preference formation takes place [19], the SSB availability total effect on a cohort's adulthood obesity might actually be much bigger than what we have witnessed from children and adolescent samples. 

This study is limited in that the estimation was done in a cross-sectional dataset. Even though the propensity score matching method helps deal with the selection bias issue, it will be ideal if we can work with longitudinal datasets covering SSB consumption before and after the soda ban at schools. Moreover, as children and adolescents might replace their SSB intake with other kinds of beverage after a restriction on their access to SSB, further studies are also needed to examine what could happen to consumption of other kinds of beverage (juice, milk, water, coffee, etc.) after those SSB bans at schools.

## Figures and Tables

**Table 1:** Descriptive statistics about variables used in the propensity score matching.

Variables	Descriptive statistics (mean or frequency distribution)
Outcome variable	

Number of SSBs on the prior day	1.09 (1.45)

Exposure variable	

Self-reported availability of sugar-sweetened beverage at school vending machines	
Yes	2285 (57.4%)
No	1698 (42.6%)

Predictors	
Age (mean)	14.4 (.026)
Gender (female)	1979 (49.1%)
Parental education	
Less than high school (referent group)	184 (21.0%)
High school only	180 (20.3%)
Some college	189 (21.3%)
Graduated from college	334 (37.7%)
Race/ethnicity	
White (referent group)	70 (7.9%)
African American	176 (4.4%)
Latino	823 (20.7%)
Asian	321 (8.1%)
School type (public schools)	3505 (87.0%)
Household income (at or above federal poverty line)	3490 (87.6%)

*N* = 3983	

**Table 2:** Probit regression predicting the propensity scores of sugar-sweetened beverage's availability at school vending machines.

Predictor	Probit coefficient
Gender (female = 1)	0.06
Age	2 .33***
Age square	−0.07***
Parental education	
Less than high school (referent group)	
High school only	−0.03
Some college	0.07
Graduated from college	−0.11
Race/ethnicity	
White (referent group)	
African American	0.02
Latino	1.02
Asian and other	0.98
Household income (above federal poverty line = 1)	0.05
School type (attending public school = 1)	0.16**

*N* = 3983	

Coefficient significant at 10%*; 5%**; 1%***.

**Table 3:** Blockwise *t*-tests of the mean difference of propensity score between the exposure group and the control group.

Block	Mean of control group	Mean of exposure group	*P* value of the *t*-tests
[0, 0.2)	0.19	0.19	.71
[0.2,0.4)	0.27	0.26	.23
[0.4, 0.5)	0.44	0.45	.20
[0.5,0.6)	0.57	0.57	.20
[0.6,0.625)	0.61	0.61	.11
[0.625,0.65)	0.64	0.64	.73
[0.65,0.7)	0.68	0.68	.60
[0.7,0.8)	0.74	0.74	.08
[0.8,1)	0.81	0.81	.57

*N = 3983*			

**Table 4:** Blockwise *t*-tests of the Mean Difference of Sugar-sweetened Beverage Consumption on the Prior Day between the Exposure Group and the Control Group.

Block	Mean of control group	Mean of exposure group	Mean difference (standard error)
[0, 0.2)	0.32	1.11	−0.79 (0.24)
[0.2,0.4)	0.87	1.09	−0.22 (0.09)
[0.4, 0.5)	1.16	1.10	0.06 (0.14)
[0.5,0.6)	0.95	1.25	−0.11 (0.14)
[0.6,0.625)	0.97	1.26	−0.29 (0.23)
[0.625,0.65)	0.85	1.00	−0.15 (0.18)
[0.65,0.7)	1.04	1.18	−0.14 (0.18)
[0.7,0.8)	1.02	1.21	−0.20 (0.09)
[0.8,1)	1.00	.9	0.10 (0.64)

*N = 3983*			

**Table 5:** The association between SSB Availability at School Vending Machines and SSB Consumption with propensity score stratification and kernel-based matching.

	Number of Exposed	Number of control	Proportional difference using matched sample	*t*-value of the difference
Stratification	2285	1698	0.181 (.046)	3.972
Kernel-based matching	2285	1698	0.159 (.059)	2.668
